# Transcriptional Regulation of Chemokine Genes: A Link to Pancreatic Islet Inflammation?

**DOI:** 10.3390/biom5021020

**Published:** 2015-05-26

**Authors:** Susan J. Burke, J. Jason Collier

**Affiliations:** Laboratory of Islet Biology and Inflammation, Pennington Biomedical Research Center, 6400 Perkins Road, Baton Rouge, LA 70808, USA; E-Mail: susan.collier@pbrc.edu

**Keywords:** chemokine, diabetes, inflammation, islet, transcription

## Abstract

Enhanced expression of chemotactic cytokines (aka chemokines) within pancreatic islets likely contributes to islet inflammation by regulating the recruitment and activation of various leukocyte populations, including macrophages, neutrophils, and T-lymphocytes. Because of the powerful actions of these chemokines, precise transcriptional control is required. In this review, we highlight what is known about the signals and mechanisms that govern the transcription of genes encoding specific chemokine proteins in pancreatic islet β-cells, which include contributions from the NF-κB and STAT1 pathways. We further discuss increased chemokine expression in pancreatic islets during autoimmune-mediated and obesity-related development of diabetes.

## 1. Introduction

**Figure 1:**
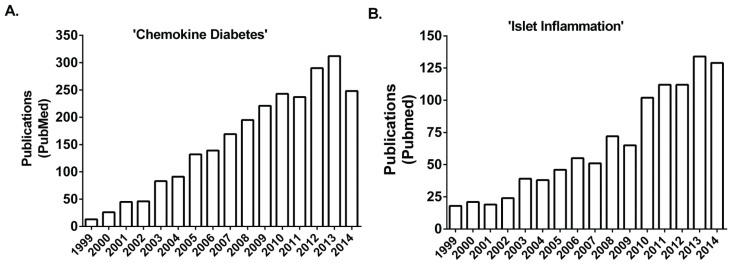
Rise in the number of publications involving chemokines, diabetes, and islet inflammation over a recent 15 year period. The number of publications (y-axis) identified using the search string shown in quotations above each graph (**A** and **B**) is shown by year (x-axis).

The dysfunction and ultimate reduction of total numbers of pancreatic islet β-cells is central to the development of both Type 1 (T1DM) and Type 2 diabetes mellitus (T2DM). T1DM is typically associated with autoimmune-mediated mechanisms that selectively eliminate the insulin-positive β-cells within the pancreatic islets [1]. The initial trigger(s) for T1DM onset is currently unknown. T2DM is correlated with obesity and is usually preceded by a period of insulin resistance and glucose intolerance prior to the loss in function islet β-cell mass that leads to the development of overt diabetes [2,3]. While the etiology of T1DM is clearly different than that of T2DM, inflammation-associated damage and dysfunction of pancreatic islet β-cells appear to be important components of each endocrine disease subtype. A common potential link between these major forms of diabetes is augmented local and systemic chemokine levels that influence the presence of leukocytes within the pancreas and within pancreatic islets (*i.e.*, insulitis). The rise in the number of publications over the last 15 years reveals the trends in research efforts related to these topics (Figure 1).

**Table 1:** Chemokines elevated in pancreatic islets during pathophysiological states.

Chemokine	Chemokine Receptor	Immune Cell Recruited	Elevated in Islets During	References
CCL2	CCR2	Monocyte/macrophage/T-lymphocyte	Chemical Induction, Cytokine Exposure, T1DM, T2DM	[4,5,6,7,8,9,10,11,12,13]
CXCL1	CXCR2	Neutrophil	Cytokine Exposure, T1DM, T2DM, Viral Infection	[6,11,13,14,15]
CXCL2	CXCR2	Neutrophil	Cytokine Exposure, T1DM, T2DM	[6,13,14,16]
CXCL10	CXCR3	T-lymphocyte	Chemical Induction, Cytokine Exposure, T1DM, T2DM, Viral Infection	[10,13,15,17,18,19,20,21]

Chemical induction includes: Alloxan, high-dose streptozotocin, and/or multiple low-dose streptozotocin injection. Cytokine exposure includes IL-1β, IFN-γ, TNF-α, or combinations of these molecules. T1DM, Type 1 diabetes mellitus; T2DM, Type 2 diabetes mellitus.

## 2. Islet-Derived Chemokines in T1DM and T2DM

Pancreatic islets isolated from various rodent models of diabetes reveal the expression of genes linked to immune cell recruitment and β-cell dysfunction; islets isolated from human diabetic subjects show similar elevated gene expression patterns (Table 1). In addition, isolated rodent and human pancreatic islets exposed to either cytokines or elevated lipid levels display an increase in a variety of inflammatory markers, which includes elevated expression of specific genes linked to leukocyte recruitment. While the ability of pro-inflammatory cytokines (e.g., IL-1β and IFN-γ), and excess lipid (e.g., palmitate) to suppress glucose-stimulated insulin secretion has been observed for more than a decade [22,23,24], less is known about the transcriptional reprogramming associated with inflammatory stimuli that lead to losses in islet β-cell function and mass.

One major class of genes, which are rapidly activated in response to pancreatic β-cell exposure to inflammatory stimuli, encode the chemotactic cytokine (aka chemokines) family of soluble, secreted, signaling proteins. Increasing the production and cellular release of specific chemokines influences immune cell movement and immunological activity. Once secreted, chemokines influence both innate and adaptive immune cell recruitment and activity by signaling through specific G-protein coupled receptors (GPCRs). For example, inducing β-cell damage using the multiple low dose streptozotocin protocol promotes global chemokine production within pancreatic islets, which leads to leukocyte recruitment, β-cell death, and hyperglycemia [4]. Additionally, several chemokines are elevated in the circulation of rodents and humans during diverse pathophysiological states (Table 2).

The chemokines are separated into four categories based on the positioning of their first cysteine residue: C chemokines have no adjacent Cys, while C-C chemokines have the first two Cys residues (out of four total) adjacent to each other; the C-X-C and CX_3_C chemokines contain intramolecular cysteine residues separated by one or three amino acids, respectively [25]. The C-C subfamily is the largest, and together with the C-X-C family, controls a large portion of leukocyte migration during innate and adaptive immune responses [26,27]. We note that all chemokine genes expressed in rodent and human pancreatic β-cells that we have examined thus far contain genomic elements recognized by the NF-κB family of transcription factors. This is in agreement with other bioinformatics based approaches [28]. Many chemokine genes also contain either gamma activated sequences (GAS), interferon-stimulated response elements (ISREs) or both types of genomic response elements within their proximal gene promoter regions. Signals that activate NF-κB (e.g., IL-1β, TLR2/4 ligands, *etc.*) and Type I and II interferons are associated with islet inflammation and β-cell death and dysfunction [29,30,31]. Thus, in our view, a broad understanding of pro-inflammatory signal integration at chemokine gene promoters (e.g., in response to IL-1β and IFN-γ) is essential to understanding inflammation in general and for identification of new targets for therapeutic intervention.

**Table 2:** Chemokines elevated in circulation during pathophysiological states.

Chemokine	Elevated in Circulation During	References
CCL2	Obesity, T1DM, T2DM	[32,33,34,35,36]
CXCL1	Obesity, T1DM, T2DM	[18,37,38]
CXCL2	Obesity	[39]
CXCL10	T1DM, T2DM, Viral Infection	[37,40,41,42,43,44]

T1DM, Type 1 diabetes mellitus; T2DM, Type 2 diabetes mellitus.

Transcriptomic technologies (e.g., oligonucleotide microarrays, RNA-sequencing, *etc.*) have yielded novel insights into global changes in gene expression patterns in normal pancreatic islets (*i.e.*, non-diseased states) *versus* inflammatory, pathological states [6,7,45]. As shown in Table 1, a commonality in various models of disease revealed by transcriptomic studies is that multiple genes encoding chemokines are markedly upregulated in pancreatic islets from various disease models and from islets exposed to either pro-inflammatory cytokines (e.g., IL-1β, IFN-γ, *etc.*) or to fatty acids (e.g., palmitate). Islets isolated for transplantation also display patterns of gene expression that are indicative of inflammation, including increased expression of chemokine genes [46]. This is important because immune cell infiltration and/or increased immune cell activity within pancreatic islets or islets at extra-pancreatic sites have been documented in the following situations: islet transplantation, palmitate infusion, STZ-induced β-cell injury, T1DM, T2DM, and viral infection.

The most likely biochemical reason for infiltration of leukocytes into a tissue is the secretion of a chemoattractant molecule that induces movement of such cells into a site of inflammation [47]. Therefore, it is plausible to postulate that a series of soluble factors (e.g., chemokines) synthesized and secreted directly from β-cells contribute to early and sustained leukocyte modulation via their cognate receptors. Consequently, the insulitis seen in rodents and humans with diabetes mellitus may be a consequence of direct islet β-cell production of chemokines. The transcriptional regulation of specific chemokine genes in pancreatic β-cells exposed to pro-inflammatory stimuli is therefore considered below.

## 3. Transcriptional Regulation of Individual Chemokine Genes in Pancreatic β-Cells

Multiple immune cells make and secrete IL-1β, including dendritic cells, monocytes, macrophages, and neutrophils. In addition, neutrophil expression of the IL-1β gene is enhanced in response to a migratory signal [48]. T-cells, which produce IFN-γ, are essential for the development of diabetes in non-obese diabetic (NOD) mice [49] and in humans [50,51]. Thus, examining the transcriptional responsiveness of the islet β-cell to these cytokines offers numerous insights into inflammation-associated dysfunction and islet-immune cell interactions.

### 3.1. Chemokine C-C Motif Ligand 2 (CCL2 Aka Monocyte Chemoattractant Protein-1/MCP-1)

CCL2 is a powerful signal promoting recruitment of various immune cells (e.g., monocytes and macrophages) into secreting tissues, including pancreatic islets [52,53]. The CCL2 gene is regulated positively by IL-1β and negatively by glucocorticoids [5]. Importantly, CCL2 co-localizes with insulin in pancreatic islets, demonstrating β-cells are capable of direct chemokine production [8]. Increasing the secretion of CCL2 from islet β-cells influences monocyte and macrophage recruitment and activity via CCR2 [52]. CCL2 is induced by IL-1β in the clonal 832/13 and INS-1E β-cell lines, two distinct rat insulinoma cell culture models, based on our unpublished microarray experiments [54]. In addition, CCL2 expression is increased in rat and human islets exposed to IL-1β [5] and in islets isolated from a human subject with T1DM [9]. The elevated expression of CCL2 in response to β-cell exposure to IL-1β requires the p65 subunit of NF-κB [5]. CCL2 has a baseline expression in β-cells, indicating it may have both homeostatic and inflammatory actions. By contrast, expression of the CCL20 gene, encoding a distinct chemokine, is largely undetectable in healthy islets and clonal β-cell lines that have not received an inflammatory stimulus. However, CCL20 is highly inducible by pro-inflammatory signals, such as IL-1β or palmitate, in rodent and human islets, and in rodent β-cell lines [7,32], indicating responsiveness in specific settings. For example, both CCL20 and CCL2 are elevated in circulation during obesity [32,55], a condition associated with chronic, low-grade inflammation.

IL-1β-dependent increases in CCL2 gene expression, promoter activity and secretion are decreased by low molecular weight inhibitors of the p38 MAPK but not by inhibitors of ERK and JNK kinases [5]. Furthermore, the inhibitor of nuclear factor kappa-B kinase subunit beta (IKKβ), which is located upstream of p65 in the NF-κB signaling pathway, is also involved in regulating expression of the CCL2 gene. Inhibition of IKKβ with the small molecule 2-[(Aminocarbonyl)amino]-5-(4-fluorophenyl)-3-thiophenecarboxamide (TPCA) prevents IL-1β-mediated expression in β-cell lines while overexpression of a constitutively-active IKKβ drives CCL2 mRNA synthesis and protein secretion [5]. These *in vitro* data are consistent with transgenic overexpression of constitutively-active IKKβ, which also leads to elevated CCL2 expression in islets *in vivo* [56].

The CCL2 gene contains bona fide NF-κB responsive elements that control gene expression in response to inflammatory signals. Preventing nuclear translocation of the primary DNA binding NF-κB transcription factor subunit p65 blocks the IL-1β-mediated increases in CCL2 gene transcription [5]. Specific and targeted deletion of p65 protein by siRNA delivery also prevents CCL2 induction by IL-1β, while siRNA-directed decreases in the p50 subunit do not have this effect [5]. Moreover, Ser276, within the Rel homology domain of p65, is critical for IL-1β to increase production of CCL2. Thus, the p65 subunit of NF-κB is the major transcription factor used to induce the expression of the CCL2 gene in pancreatic β-cells, leading to mRNA accumulation and secretion of CCL2 protein [5]. As proof of principle for inflammatory disease, elevated expression of CCL2 and other chemokines is observed in islets of NOD mice [10], hIAPP transgenic mice [57,58], obese mice [11,32], humans with T1DM [9], and humans with T2DM [12]. Notably, these are all situations where IL-1β levels are also enhanced.

### 3.2. Chemokine C-X-C Motif Ligands 1 and 2 (CXCL1/CXCL2)

Rodent and human islets and pancreatic β-cell lines all markedly upregulate CXCR2 ligands (e.g., CXCL1, CXCL2, *etc.*) in response to inflammatory signals [13,16]. Moreover, circulating CXCL1 is elevated in human T1DM [38], obese rodents [59], and human subjects with T2DM [37]. Islets from obese mice [11] and mice transgenic for hIAPP [57] also have higher expression of CXCL1 relative to their control counterparts. Both local and systemic CXCL1 bind to the CXCR2 receptor, which is highly abundant on neutrophils. CXCR2 likely participates in, or directly controls, neutrophil recruitment and the associated inflammation-mediated destruction of pancreatic islet β-cells [60,61]. Indeed, systemic delivery of compounds that inhibit the activity of the receptors CXCR1 and CXCR2 augments islet graft function after transplantation [61], and protects against autoimmune forms of diabetes in mice [60]. These findings implicate CXCR1^+^ and CXCR2^+^ immune cells (e.g., neutrophils) as major contributors to the process of islet β-cell destruction. In addition, pharmaceutical targeting of CXCR2 for several other inflammatory diseases is currently being considered [62,63,64].

In pancreatic β-cells, the genes encoding CXCL1 and CXCL2, which are both ligands for CXCR2, are strongly and rapidly regulated by signals that activate NF-κB [16]. In clonal β-cell lines, rat islets and human islets, CXCL1 and CXCL2 are primary response genes (*i.e.*, they are activated rapidly in a manner that does not require new protein synthesis). Despite the presence of sites recognized by STAT1 transcription factors, the CXCL1 and CXCL2 genes do not respond to IFN-γ alone as a transcriptional activation signal. Instead, these genes use STAT1 as an accessory factor to support the transcriptional response induced by IL-1β activation of the NF-κB p65 and p50 heterodimer [16]. While the increase in transcription of the CXCL1 and CXCL2 genes in response to IL-1β is blocked by inhibition of IKKβ, interfering with the p38 MAPK has no effect [16]. In addition, specific covalent modifications to histone H3 within the core promoter of the CXCL1 and CXCL2 genes are produced upon β-cell exposure to IL-1β. These include increased acetylation of Lys14 and methylation of Lys4 and decreased methylation of both Lys9 and Lys27 [16]. These coordinated alterations in histone chemical modifications correlate with an increase in Ser5 phosphorylation of RNA polymerase II (Pol II) carboxy terminal domain (CTD) within the core promoter of the CXCL1 and CXCL2 genes. Furthermore, phosphorylation of Ser2 within the RNA Pol II CTD is enhanced on the coding region of the CXCL1 and CXCL2 genes after cellular exposure to IL-1β, consistent with elongation of transcription. Taken together, the post-translational modifications to RNA Pol II are congruent with the appearance of transcripts encoding chemokines within 1 h after IL-1β stimulation and an increase in chemokine protein secretion by 3 h [16].

The CXCL1 and CXCL2 proteins have potent biological effects on human peripheral blood neutrophils, which include increased migration and alterations in cell surface integrin expression. Human neutrophils increase their surface expression of the integrins Cd11b and Cd11c, but not Cd11a, after exposure to either CXCL1 or CXCL2 [16]. The combination of both chemokines is redundant in this context. The pathophysiological significance of these findings is postulated to be as follows: islet β-cells exposed to cytokines, such as IL-1, synthesize micromolar levels of nitric oxide (NO•) [31,32,33,34] while concurrently synthesizing and secreting ligands that chemoattract neutrophils. CXCR2 activation by CXCL1 or CXCL2 enhances neutrophil integrin expression, which allows for neutrophil adherence to endothelial cells to facilitate migration into inflammatory sites. In addition, elevated levels of NO• produced by β-cells likely inactivate myeloperoxidase (MPO), a protein secreted by neutrophils [65]. Inactive MPO promotes macrophage production of cytokines [66]. Thus, neutrophils may be assisting the resident macrophage-mediated destruction of islet β-cells via production of CXCR2 ligands; the CXCR2 ligands CXCL1 and CXCL2 are upregulated by β-cells as primary response genes. In addition, neutrophil-derived MPO, inactivated in response to either β-cell or macrophage production of NO, likely enhances cytokine production from macrophages. Collectively, these signals may be initiating, contributing to, and/or maintaining an immunological response within the pancreas.

### 3.3. Chemokine C-X-C Motif Ligand 10 (CXCL10)

Because the mechanism(s) underlying antigen-specific leukocyte migration into the pancreatic islets is currently unknown, a systematic analysis of the expression and function of individual chemokines in response to pro-inflammatory cytokines, such as IL-1β and IFN-γ, is an essential component for understanding islet inflammatory processes that lead to diabetes mellitus. The gene encoding CXCL10 is responsive to both IL-1β and IFN-γ in pancreatic β-cells, with synergistic expression when both cytokines are present [17]. The p65 and p50 subunits of NF-κB are recruited to the κB genomic response elements in the CXCL10 proximal gene promoter within 15 minutes after β-cell exposure to IL-1β.

IFN-γ is a key regulatory cytokine that participates in β-cell demise [67,68] and induces both the expression and phosphorylation of STAT1 proteins [69]. STAT1α is a versatile regulator of various genes involved in β-cell death and dysfunction [70] and is one of the factors that potentiates the IL-1β-mediated induction of various genes in pancreatic β-cells [16,17,71,72], including CXCL10. Circulating levels of CXCL10 are also elevated in T1DM and T2DM (Table 2). Therefore it is feasible that chemokine release from pancreatic β-cells, in response to pro-inflammatory signals, is a contributing factor to the observed increases in circulating chemokine levels.

Analogous to what is observed for NF-κB transcription factors in response to IL-1β, STAT1 is also observed at ISRE containing regions of the CXCL10 gene promoter after IFN-γ exposure. These responses coincide with recruitment of the coactivators p300 and CBP [17]. Interfering with JAK signaling using a low molecular weight inhibitor prevents the cytokine-mediated increases in CXCL10 mRNA and promoter activity. In addition, adenoviral delivery of STAT1 with Phe substituted for Tyr at position 701 decreases gene activation and release of CXCL10 protein [17]. Interestingly, a replacement of Ser727 with either Ala or Thr has no effect on cytokine-mediated expression of the CXCL10 gene [17]. Moreover, and consistent with rapid upregulation of chemokine gene transcription by pro-inflammatory stimuli, *in vivo* expression of a regulatory protein that blocks NF-κB transcriptional activity prevents insulitis and hyperglycemia that normally occurs in mice after injections of multiple low doses of streptozotocin. Part of the mechanism for this protection includes decreased expression of CXCL10 [21]. Thus, NF-κB and STAT1 cooperate to control the production of CXCL10 in pancreatic β-cells exposed to pro-inflammatory signals, which in turn influences immune cell activity within the pancreas.

CXCL10 is closely associated with autoimmune-mediated diabetes [20,73], and the expression of the CXCL10 gene increases prior to onset of diabetes in NOD mice, a rodent model of autoimmunity [42]. The CXCR3 receptor, activated by multiple ligands including CXCL10, is present on T-lymphocytes [74]. CXCR3 is present on both CD4^+^ and CD8^+^ T-cell populations and is associated with a Th1 phenotype. Islet β-cells make CXCL10 under several distinct inflammatory conditions (Table 1). CD4^+^ T-cells are found in pancreatic islets in both T1DM and T2DM [75] and are also present in the exocrine tissue of subjects with T1DM [76]. CD8^+^ T-cells are also present in islets of individuals with T1DM [75]. Arrival of T-cells into pancreatic tissue before and during disease onset and progression may be due, at least in part, to chemokine molecules (e.g., CXCL10) released from β-cells after exposure to pro-inflammatory stimuli.

## 4. Conclusions

Inflammation is associated with tissue dysfunction in various human diseases, including T1DM and T2DM. Inflammatory signaling pathways are inducible within pancreatic islets, promoting alterations in tissue function. If this inflammation does not resolve, eventual losses in overall function and total numbers of islet β-cells leads to overt hyperglycemia, *sine qua non* for diagnosis of diabetes mellitus. The pancreas is constantly accessible to immune cells [77] and the islet β-cells synthesize chemokines that recruit most, if not all, types of leukocytes. Importantly, innate immune cells, such as macrophages and neutrophils, are also capable of secreting chemokines that support leukocytic infiltration [78,79]. Thus, insulitis is a carefully coordinated event between the tissue being infiltrated (e.g., islets) and the immune cell populations participating in the response.

Because leukocytes enter pancreatic islets during various pathophysiologic states, we propose that regulatory signals, such as chemokines, produced by endogenous islet β-cells in response to an inflammatory stimulus (e.g., IL-1β) produce two possible outcomes: (1) a feed-forward cycle of immune cell recruitment and activity that ultimately results in β-cell death and dysfunction; (2) Influencing the activity of leukocytes already present in either endocrine or exocrine tissues. These two possibilities are not mutually exclusive.

Islet β-cell production of chemokines is fitting with insulin-positive islets being more strongly associated with insulitis [80,81]. It is thus possible that the inflammatory actions of immune cells are targeted against the β-cells themselves rather than islets in general, which is consistent with data demonstrating that specific genes expressed in β-cells are markers of autoimmune diabetes [82,83]. Because cells of the innate and adaptive immune system are linked to T1DM [14,84,85], obesity [86,87,88] and T2DM [12,75], this indicates an overall importance for understanding signals and mechanisms associated with the production of immunomodulatory proteins, such as the chemokines, produced by islet β-cells. Additionally, the pancreatic islets may be impacted by an overall increase in local and/or systemic mediators of inflammation, including in response to viral infection [15], and thus produce chemokines in order to inform the immune system of their response to such stimuli.

Therapies to prevent immunological responses targeted against pancreatic islets have thus far included various approaches, such as the targeting of specific enzymes, e.g., HDACs [89] and IKKs [90,91]. Additionally, antibody-based and low molecular weight inhibitors of chemokine receptors have been suggested as therapeutics to prevent rejection of transplanted islet tissue [92]. Interference with cell surface receptors, such as the IL-1R, has shown promise towards improving islet β-cell function [93,94]. Novel ligands that activate the intracellular receptors, (e.g., NR3C1 aka glucocorticoid receptor; NR1C3 aka PPARγ, *etc.*) may also have a future role in suppressing inflammatory responses that impair islet β-cell mass and function [95,96,97]. The use of various botanicals, including polyherbal combinations delivered via the diet [98], also show promise as direct treatments and/or as adjuvants to existing therapeutic strategies. The most efficacious of these approaches remains to be determined.

In summary, the production of various chemokines from islet β-cells exposed to pro-inflammatory stimuli, such as cytokines and lipids, likely influences leukocyte activity and infiltration into the pancreas and more specifically into the islets of Langerhans. A thorough understanding of how this class of soluble secreted immunomodulatory proteins, and their cognate receptors, influence immunological responses that contribute to metabolic disease could lead to promising new strategies to prevent or reverse the defects in tissue function associated with the development and progression to diabetes mellitus.
